# Multiple Giant Cell Tumors of Tendon Sheath Found within a Single Digit of a 9-Year-Old

**DOI:** 10.1155/2016/1834740

**Published:** 2016-08-10

**Authors:** John S. Hwang, Valerie A. Fitzhugh, Peter D. Gibson, Jacob Didesch, Irfan Ahmed

**Affiliations:** ^1^Department of Orthopaedic Surgery, Rutgers, The State University of New Jersey, New Jersey Medical School, Newark, NJ 07103, USA; ^2^Department of Pathology and Laboratory Medicine, Rutgers, The State University of New Jersey, New Jersey Medical School, Newark, NJ 07103, USA

## Abstract

Giant cell tumor of tendon sheath is one of the most common soft tissue tumors of the hand. These tumors typically occur in the third or fourth decade of life and present as solitary nodules on a single digit. Currently, the greatest reported number of lesions found within a single digit is five. Although uncommon, giant cell tumor of tendon sheath does occur in the pediatric population. Herein we present a report of a rare case of GCTTS in a child in which seven lesions were identified within a single digit—the greatest number of lesions within a single digit reported to date.

## 1. Introduction

Giant cell tumor of tendon sheath (GCTTS) is the second most common soft tissue tumor of the hand, second to ganglion cyst [1–3]. This tumor is also known as localized tenosynovial giant cell tumor. These tumors were previously described as localized nodular synovitis, fibrous xanthoma, or pigmented villonodular tenosynovitis. Furthermore, GCTTS is histologically identical to pigmented villonodular synovitis (PVNS), with the only distinction being the location of the masses; GCTTS is located within a tendon, while PVNS is an intraarticular lesion. Typically, GCTTS occurs within the ages of 30–50 and affects women twice as often as men [3].

Though uncommon, GCTTS has been seen in the pediatric population. A case series performed by Gholve et al. demonstrated that GCTTS in the pediatric population behaves similarly to the adult lesion [4]. These lesions are often solitary within a single digit, but cases have been reported in which multiple lesions were identified within a single digit. Currently, the greatest number of lesions reported in a single digit was five [5]. Herein we present a report of a rare case of GCTTS in a child where seven lesions were identified within a single digit, the most reported to date.

Informed consent was obtained from the patient's guardian for print and electronic publication.

## 2. Case Presentation

**Figure 1:**
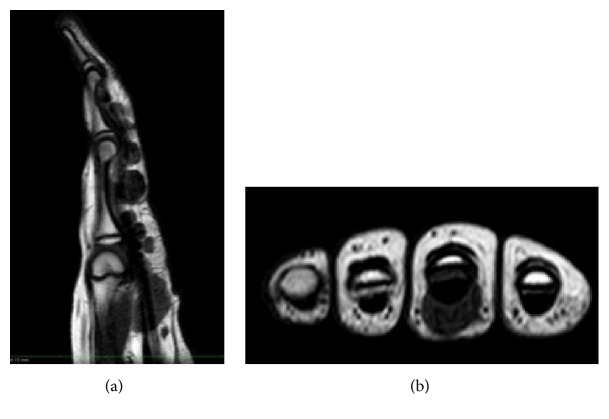
(a) MRI T1 sagittal image demonstrating hypodense masses on the volar aspect of the middle finger. (b) MRI T1 axial image demonstrating hypodense masses on the volar aspect of the middle finger.

A 9-year-old female, right hand dominant, presented to our institution with a one-year history of left middle finger pain and palpable growing masses. The patient was first evaluated by her pediatrician. Plain radiographs were performed by her pediatrician and the report stated no significant findings. Her past medical history is significant for hypothyroidism which is being treated with levothyroxine. Magnetic resonance imaging (MRI) was performed to further evaluate the masses. The study demonstrated multiple hypodense masses and seven identifiable masses, on the volar aspect of the proximal, middle, and distal phalanx of the left middle finger (Figures 1(a) and 1(b)).

**Figure 2:**
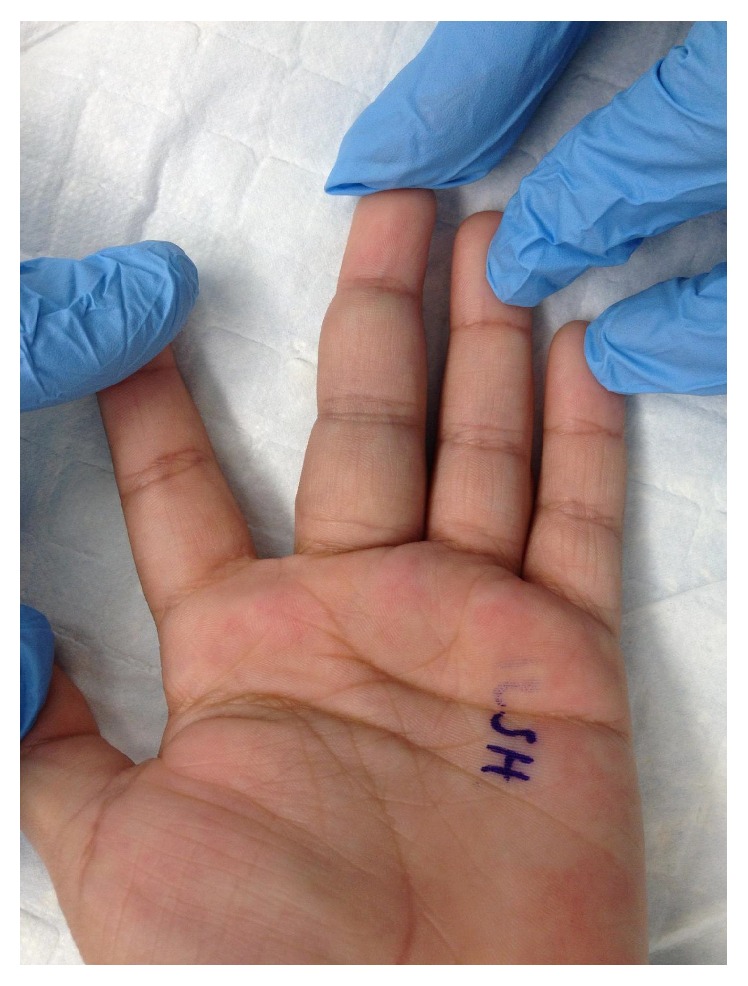
Preoperative image of left hand demonstrating fullness of the middle finger with bluish discoloration.

Three months after obtaining the MRI, the patient was seen in the clinic for evaluation by the orthopaedic hand service. Physical examination revealed mild swelling and tenderness throughout her left middle finger; however, no sensory deficits were noted, and brisk capillary refills were present. Three palpable small masses could be felt throughout the volar aspect of her finger. Bluish discoloration could be seen over some of these masses (Figure 2). The range of motion of her finger was significantly limited due to pain and swelling: 0–10 degrees in proximal interphalangeal joint, 0–15 degrees in distal interphalangeal joint, and 0–30 degrees in metacarpophalangeal joint. There were no enlarged lymph nodes found on physical examination. The patient denied recent weight loss, fevers, chills, fatigue, or trauma. A decision was made to perform an excisional biopsy to identify the masses through histological examination.

**Figure 3:**
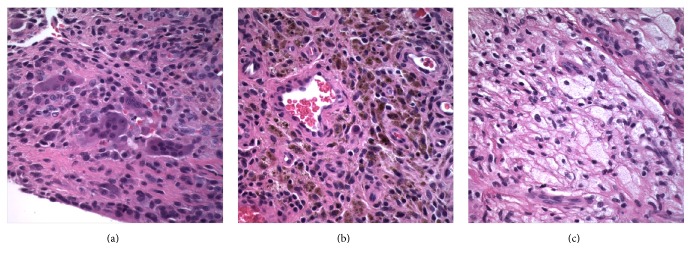
(a) Hematoxylin and eosin stained section demonstrating giant cells and mononuclear cells embedded within a collagenous matrix (600x). (b) Hematoxylin and eosin stained section demonstrating hemosiderin laden cells (600x). (c) Hematoxylin and eosin stained section demonstrating clusters of xanthomatous cells (600x).

**Figure 4:**
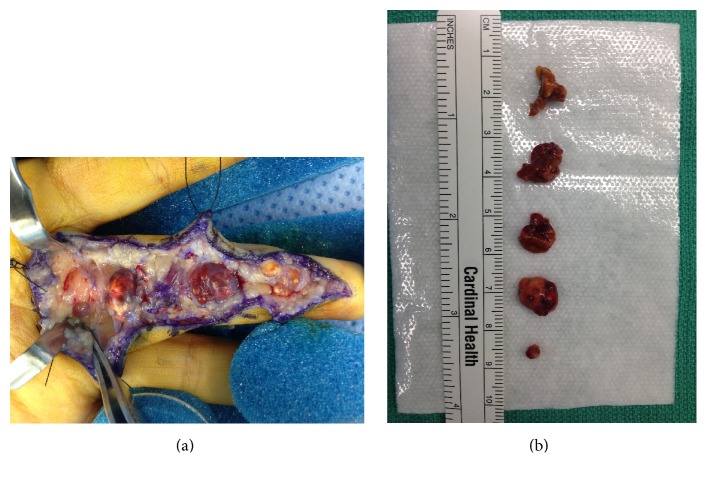
(a) Intraoperative image demonstrating five masses within the flexor tendon sheath of the middle finger. (b) Gross pathology demonstrated five resected masses found within the flexor tendon sheath of the middle finger.

The patient initially underwent excisional biopsy of two of the masses that were abutting each other. Definitive diagnosis was not obtainable from the initial frozen section. The third mass was located more proximal and a decision was made to not excise this lesion until a definitive diagnosis could be made. Tissue was submitted for further histological examination. Standard sections revealed a cellular process including giant cells and mononuclear stromal cells within a collagenous matrix (Figure 3(a)). Hemosiderin deposition and clusters of xanthomatous cells were also identified (Figures 3(b) and 3(c)). These findings were consistent with GCTTS. Two months after the initial surgery, a decision was made to excise the remaining masses. Five additional lobular masses were identified intraoperatively and resected (Figures 4(a) and 4(b)). Permanent sections from the remaining masses were consistent with GCTTS and histologically analogous to the previous biopsy.

**Figure 5:**
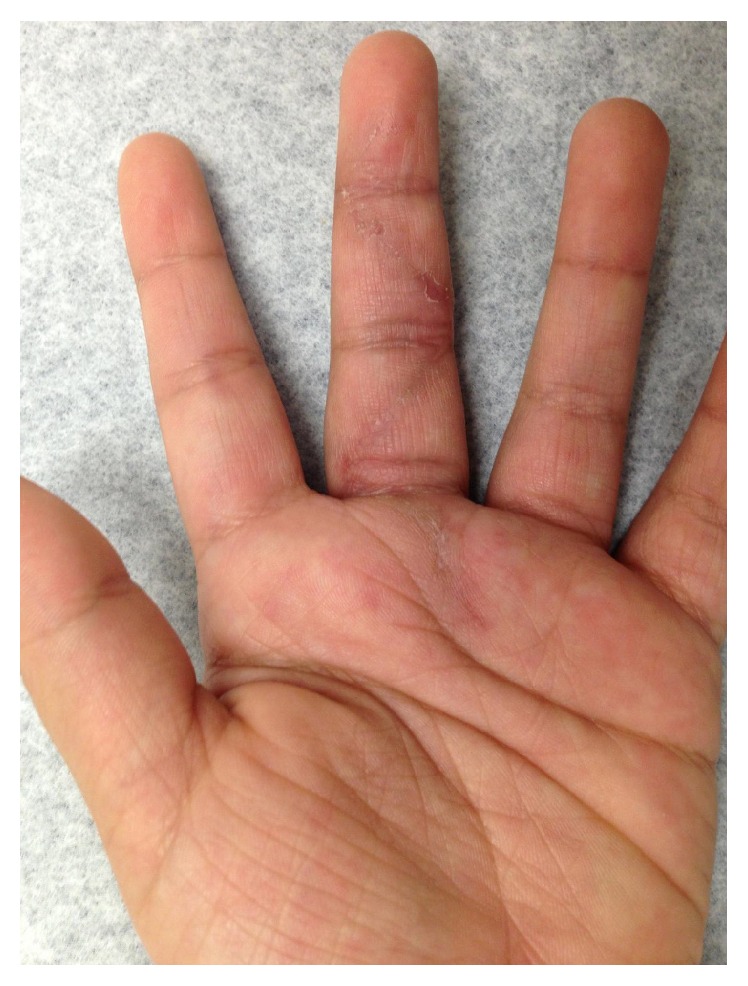
Healed incision of the left hand.

At this time, the patient is two years from her second surgery. She denies any pain and has full range of motion of her left middle finger. Her incisions are well healed and no foci of recurrence are noted at this time (Figure 5).

## 3. Discussion

GCTTS is a common benign soft tissue tumor found in the hand which originates in the synovium of the flexor sheath. They typically go through stages of dormancy and increased activity making these lesions suddenly symptomatic or noticeable. Although they most often occur in the hand and fingers, these tumors can also occur in larger joints like the feet, ankles, knees, and elbow. In the hand, they typically occur adjacent to the distal interphalangeal joint of the index or long finger.

With recurrence rates reported up to 45% [6], careful attention must be paid when excising these tumors. A systematic review study performed by Fotiadis et al. examined possible risk factors for recurrence [7]. They stated that poor surgical technique with incomplete excision increased the risk of recurrence. The authors found that careful dissection and excision with magnification equipment resulted in the lowest recurrence rate. Additional risk factors for recurrence included location at distal interphalangeal joint of finger, osseous pressure erosion, mitotic activity on histology, proximity to arthritic joint, gene nm 23, and Al-Qattan type II tumors.

In 2001, Al-Qattan described a classification system for GCTTS. The classification systems were macroscopically divided into two main types, tumor with or without a single pseudocapsule [2]. The patient in this study had seven discrete lesions within one digit. According to this study, our patient is classified as having a Type IIC tumor, which is multicentric type with separate discrete lesions in the same digit. Type II tumors, ones which are not surrounded by one pseudocapsule, were found to have a recurrence rate of 38%. In our case, our patient currently has not had a recurrence with one-year follow-up.

Our case also represents the largest number documented discrete GCTTS lesions in a single digit in current literature. Singh et al. reported a case in an adult with five lesions within a single digit [5]. That patient had complete excision of the lesion and no recurrences. In the study by Al-Qattan, one patient was found to have Type IIC classification with a history of recurrence.

In 2007, Gholve et al. published the largest case series of GCTTS in the pediatric population [4]. The researchers found the rate recurrence in their retrospective review of 29 children to be 0%. Although the mean follow-up was four years, they state that meticulous dissection with magnification equipment may assist in decreasing the rate of recurrence. Other studies have also made similar recommendations in preventing recurrences of GCTTS [1].

In conclusion, our case represents a rare case of GCTTS in a child with seven lesions found within a single digit. To our knowledge, this is the first case where more than five solitary lesions were found within a single digit. In addition, these lesions were found in a child which is even less typical. Due to the relatively high rate of recurrence found with these tumors, especially those with multiple solitary lesions, complete and meticulous resection of these tumors with magnification equipment is recommended.
